# Antibiotic prescription pattern in a Ghanaian primary health care facility

**DOI:** 10.11604/pamj.2017.28.214.13940

**Published:** 2017-11-07

**Authors:** James Prah, Joseph Kizzie-Hayford, Emmanuel Walker, Adelaide Ampofo-Asiama

**Affiliations:** 1 University of Cape Coast Hospital, University of Cape Coast, Ghana

**Keywords:** Rational drug use, prescribing indicators, patient indicators, University of Cape Coast Hospital, Ghana, antibiotic prescription

## Abstract

**Introduction:**

A major challenge to the provision of health care worldwide is the irrational use of antibiotics. To help promote rational use of drugs, standard treatment guidelines (STG) and essential medicine lists and facility-specific formularies have been developed to be used by clinicians. This study assessed the prescription pattern of antibiotics and explored the use of STG by clinicians.

**Methods:**

A prospective cross sectional study that made use of seven core drug use indicators was conducted from February, 2017 to July, 2017. Prescribing indicators were assessed using 388 prescriptions that were submitted for filling and dispensing at the pharmacy unit of the hospital. Clinicians were interviewed to assess their use of STG. Data were analyzed using SPSS version 20.0 with a p-value of < 0.05 considered as statistically significant.

**Results:**

A total of 1351 drugs were prescribed for 388 patients. The average number of medicines per prescription was 3.5. Of the 388 prescriptions, 55.2% bore antibiotics, with amoxicillin (22.5%) and ciprofloxacin (18.4%) being the most prescribed antibiotics. Patients' knowledge about their medications was found to be significantly associated with the number of drugs per prescription (p = 0.001), age (p = 0.015) and educational level (p = 0.001). Only 41.7% of prescribers had copies of STG and used them.

**Conclusion:**

The prescribing and dispensing practices in the hospital were generally not satisfactory with a low patronage of STG among prescribers. In order to improve the situation, clinicians should practice evidence based medicine rather than empirical treatment of conditions as well as use the STG in practice.

## Introduction

Antibiotics, also known as antibacterials are antimicrobials that are used in the prevention and treatment of bacterial infection [1]. They act by either selectively killing or inhibiting the growth of disease-causing bacteria. Ever since antibiotics were introduced into medicine in the 1940s [2], antibiotics have revolutionalized healthcare. In recent times however, the once treatable infections are becoming difficult to cure, therefore raising cost of healthcare leading to rise in mortality with costs to both individuals and society [2]. Many pathogens are now resistant to more than one antibiotic and the new, last resort antibacterials are more expensive and cannot be obtained by those who need them [2]. The World Health Organization (WHO) classified antibacterial resistance as a serious threat that is happening right now in every region of the world and has the potential to affect anyone, of any age and in any country [3]. The United States Centers for Disease Control and prevention (CDC) estimates that antibiotic resistance is responsible for more than 2 million infections and 23,000 deaths each year in the United States, at a direct cost of $20 billion [4]. In some African studies conducted in Tanzania and Mozambique, resistant infections were found to have resulted in increased mortality in neonates and children under five [5, 6]. The emergence of resistant pathogens has been attributed to irrational prescriptions and use of antibiotics, polypharmacy and use of substandard drugs [7].

In 1993, the world health organization introduced the manual “how to investigate drug use in health facilities” [8]. This manual provides a methodology for obtaining reproducible and objective measures of the effectiveness and efficiency of use of drugs in health facilities [9]. Several studies conducted across the globe in low, middle and high income countries have revealed inappropriate antibiotic use, which correlates positively with antibiotic resistance development [10]. The Ministry of Health in Ghana has developed an essential drug list and Standard Treatment Guidelines (STGs) to guide prescription practices in Ghana [11]. However, the adoption of these guidelines by prescribers is voluntary [12]. Several studies within Ghana [13, 14] and outside Ghana [15, 16] have shown varying degrees of inappropriate prescriptions and use of drugs in public health facilities. These studies have been across all levels (primary, secondary and tertiary) of health care with varying levels of antibiotic prescription patterns ranging from a low of 11.9% to a high of 60.7%. A study conducted at the Cape Coast Teaching Hospital which is a tertiary level hospital [17], showed that 18.9% of all prescriptions contained antibiotics. Even though studies conducted in other parts of Ghana [12, 18] showed that the level of antibiotic use in primary health-care facilities was high and inappropriate, there have been no well-structured study at this level of healthcare in the Central Region of Ghana to investigate the antibiotic prescription patterns among prescribers and to determine their adherence to the STG recommendation for use. Regular assessment of drug prescribing practices in a health facility will help to identify specific drug use problems, sensitize clinicians on rational medicine use and provide policy makers with relevant information that could be used to review medicine-related policies in that health facility. This study therefore focuses on assessing antibiotic prescription practices in a primary health-care setting in Cape Coast, the Central Region of Ghana using selected WHO [8] rational drug use indicators.

## Methods

This was a prospective cross sectional study that was conducted at the out-patient department of University of Cape Coast Hospital from February, 2017 to July, 2017. Even though WHO (1993) recommends the use of at least 100 patient records in a single health facility for drug utilization studies, a sample size of 388 was used after calculating to obtain a minimum sample size of 384 using a national prevalence of 43.3% [14] for antibiotic prescriptions. The study made use of the WHO drug use indicators manual [8]. Seven core drug use indicators were adapted for this study. The core indicators included 5 prescribing and 2 patient care indicators. The study also used some complementary indicators of drug use performance. These were percentage of prescriptions that were in harmony with the STG, percentage of drugs actually dispensed at the pharmacy unit of the hospital and percentage of patients who went through laboratory investigations before antibiotics were prescribed for them. Using a systematic sampling technique, 388 outpatients of all ages and gender, irrespective of their medical conditions were recruited into the study as they presented their prescriptions to the pharmacy. Well trained pharmacy personnel collected the prescribing indicator data using prescriptions and consulting patients' folders for complimentary data such as age, sex, educational level, diagnoses, laboratory tests, health insurance status and relevant drug history. Other researchers interviewed patients who were 18 years and above for their understanding of their medications. All prescribers (doctors and physician assistants) who were available during the study period were interviewed using a structured questionnaire to ascertain their ownership and use of the Standard Treatment Guidelines of Ghana. The prescribing indicators used were: average number of drugs per encounter. This is to measure the degree of polypharmacy. This was calculated by dividing the total number of different drug products prescribed, by the number of encounters surveyed.

A fixed-dose combination of drugs prescribed for one case was counted as one; percentage of drugs by international nonproprietary names (generic name). This was calculated to measure the tendency to prescribe by generic name. This was determined by dividing the number of drugs prescribed by their generic names by the total number of drugs prescribed, multiplied by 100; percentage of encounters with an antibiotic prescribed. This was to measure the overall level of use of antibiotics. This was calculated by dividing the number of patient encounters (prescriptions) during which an antibiotic was prescribed, by the total number of encounters (prescriptions) surveyed, multiplied by 100; percentage of encounters with an injection prescribed. This was to measure the overall level of use injections by prescribers. It was determined by dividing the number of prescriptions which contained an injection by the total number of encounters surveyed, multiplied by 100; percentage of drugs prescribed from the hospital's essential drug lists. This was to measure the degree to which prescribing practices conform to the hospital's drug policy. It was determined by dividing the number of drugs prescribed which were listed on the hospital's essential drug list by the total number of drugs prescribed multiplied by 100. The patient care indicators were: the percentage of drugs actually dispensed. This measured the degree to which the hospital was able to provide the drugs which were prescribed. This was found by dividing the number of drugs actually dispensed at the hospital by the total number of drugs prescribed, multiplied by 100; patient's knowledge of correct doses. It measured the effectiveness of the information provided to patients on the dosage of the medicines they received. This was calculated by dividing the number of patients who were able to adequately identify and report the dosage for all drugs they received, by the total number of patients interviewed, multiplied by 100. The University of Cape Coast Institutional Review Board gave ethical approval for the study. Informed consent was obtained from each patient before being interviewed.

**Data analysis**: Data collected was entered and analyzed using the SPSS version 20. In the statistical analysis, averages and percentages were calculated for the drug use indicators. In addition, Pearson's chi-square was done to test for association between some selected variables. P-value less than 0.05 at 95% confidence interval was considered statistically significant.

## Results

**Figure 1:**
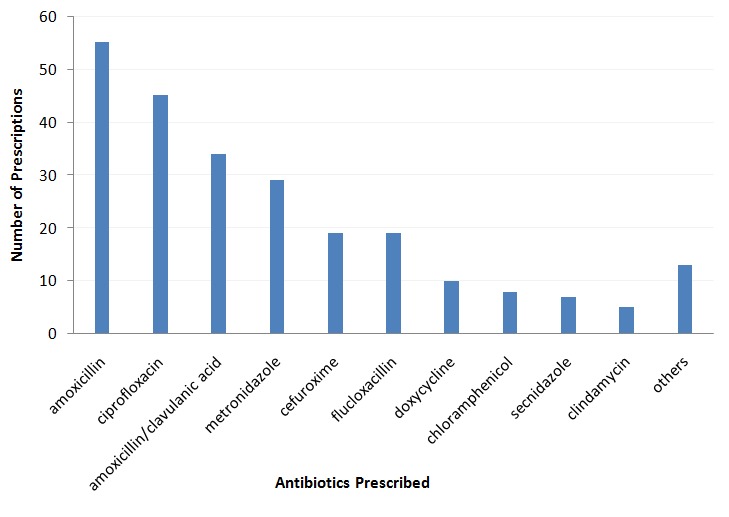
Distribution of prescribed antibiotics

A total of 388 patients with a mean age of 32.8 ± 20.6 years were involved in the study. Out of this number, 39.0% (151) were males whilst 237 (61.0%) were females. Table 1 gives details of the socio-demographic characteristics of patients. A total of 1351 drugs were prescribed on the 388 prescriptions used in the study resulting in an average of 3.5 drugs per encounter (range: 1-9). The study found that 55.2% of all prescriptions contained an antibiotic. Details of other prescribing and patient care indicators used in the study are shown in Table 2. Patients' knowledge about their medications was found to be significantly associated with the number of drugs per prescription (p = 0.002), age (p = 0.015) and educational level (p = 0.001). Sex of patients was however not significantly associated with understanding of medications (p = 0.902). The proportion of clients who received less than half of their drugs were 102 (26.3%) whilst 6 (1.5%) did not receive any of their medications. Amoxicillin (22.5%) was the most commonly prescribed antibiotic, followed by ciprofloxacin (18.4%) and amoxicillin/clavulanic acid (13.9%). Figure 1 shows the distribution of the antibiotics prescribed. A total of 40 (10.3%) of all patients were sent to perform diagnostic tests. Of the 214 patients who received prescriptions containing antibiotics, 27(12.6%) had been asked to perform investigations to confirm bacterial infections. Table 3 gives details of ownership and use of STG among prescribers and as well as other complementary indicators of drug use assessed in the study. Most of the antibiotics prescribed were to manage upper respiratory infections (29.9%), followed by urinary tract infections (19.6%), dental conditions (10.3%) and enteric fever (8.9%).

**Table 1:** Socio-demographic characteristics of patients

Characteristic	Frequency (n=388)	Percentage
**Age group (years)**		
≤18	95	24.5
19-30	102	26.3
31-60	140	36.1
>60	51	13.1
**Sex**		
Male	151	39.0
Female	237	61.0
**Health insurance status**		
Yes	370	95.4
No	18	4.6
**Level of education**		
No formal education	59	15.2
Primary	136	35.1
Secondary	94	24.2
Tertiary	99	25.5

**Table 2:** Results of core indicators of drug use and ideal WHO standards

Prescribing indicators	Result	Reference value [19,20]
Average number of drugs per encounter	3.5	< 2
Percentage of drugs by generic names	51.9%	100%
Percentage of encounters with an injection	6.4%	< 20%
Percentage of drugs prescribed from EML	85.6%	
percentage of encounters with antibiotic prescribed	55.2%	< 30%
**Patient care indicators**		
Percentage of drugs actually dispensed	69.5%	
Patient knowledge of correct dosage	88.1%	

**Table 3:** Ownership and use of STG among prescribers and other complementary indicators of drug use

Indicator	Result (%)
**Ownership of STG (n=12)**	
Yes	5 (41.7%)
No	7 (58.3%)
**Use of STG in practice (n=12)**	
Regularly	0
Occasionally	5 (41.7%)
Do not use	7 (58.3%)
**Conformity of treatment to STG**	
(n = 388)	
Yes	250 (64.4%)
No	138 (35.6%)
Percentage of drugs actually dispensed (n = 1351)	939 (69.5%)
**Patient asked to do investigations (n = 214)**	
Yes	27 (12.6%)
No	187 (87.4%)

## Discussion

The average number of drugs per encounter of 3.5 obtained in this study is well above the ideal WHO standard of less than 2 [19, 20] and findings of other similar studies worldwide conducted at different levels of healthcare [8]. It is however lower than what was found in a previous study in Ghana [21]. The high average number of drugs per encounter found in this study suggests the practice of polypharmacy and irrational drug use among prescribers. The high level of polypharmacy found in this study as compared to other studies [8, 9] could be due to a possible lack of adequate training of prescribers on rational drug use, differences in the socioeconomic profiles in the study population and a variation in the healthcare systems. This could indicate a need to educate clinicians on the risks associated with polypharmacy and irrational drug use. Such risks include increased adverse reactions, increased drug interactions and dispensing errors [21]. This finding could also be attributed probably to the treatment of multiple diseases simultaneously by prescribers. This is a documented cause of polypharmacy among prescribers [22]. Since the elderly (≥ 60 years) are likely to suffer significant co-morbidities and may therefore need multiple medications, their increasing number in any population might lead to an observed increase in the number of medicines per patient. About 13.1% of all patients in this study were ≥ 60 years higher than what pertains in the general Ghanaian population of 7.6% [23]. The 51.9% of generic prescription in this study is far lower than some previous studies by WHO in other countries that found generic prescription rates of 82-93% [8]. The generic prescription rate found in this study is well below the WHO standard of 100% [19, 20]. It is however consistent with findings of similar studies conducted in Ghana (62.6%) [21], Nigeria (55.7%) [24] and Kenya (40%) [25]. It is very important that professional bodies collaborate with regulators to influence standard practice in the African region. Fifty-five (55) percent of encounters had antibiotics prescribed. This is similar to the findings of surveys conducted in other developing countries. These studies showed that antibiotics were prescribed in 35 to 60% of all clinical encounters although an antibiotic prescription rate of less than 30% is recommended by WHO [19, 20]. Earlier studies conducted in Ghana had found that 41-55% of all prescriptions contained an antibiotic [21]. Over use of antibiotics as found in this study, may increase the risk of antimicrobial resistance, an increased cost of treatment, and the need to treat health conditions that would have previously resolved spontaneously [26]. The Mayo clinic reports that antibiotic overuse could be due to care givers prescribing antibiotics before test results confirm a bacterial infection [27].

Our study found that only 10.3% of all those who received antibiotic prescriptions actually had an investigation done to confirm diagnosis. In a study in Ghana [28], 98% of physicians stated that because of time constraints, they rarely order or never order tests. Clinicians need to be obliged to abandon empirical treatment for the more acceptable evidence-based medicine practice in order to reduce the over-prescription of antibiotics in our setting. However, there are some medical conditions that are diagnosed mainly using clinical findings. These conditions include pneumonia and upper respiratory tract infections. In the management of such conditions, clinicians usually use empirical therapy. In order to avoid the over-prescription of antibiotics, the use of standard treatment guidelines is highly recommended. The percentage of prescription with an injection encountered in this study of 6.4% is lower than the 13.4-24.1% range found by WHO in some previous studies [8]. A similar study [21] in Ghana found a higher percentage of encounter (8.3%) in which injections were prescribed. In another study that assessed prescription patterns in 12 developing countries, the percentage of injection per encounter ranged from a high of 48% in Uganda to a low of 11% in Zimbabwe [29, 30]. Indiscriminate and overuse of injections can increase the possibility of spread of blood-borne infectious diseases like HIV. An earlier study [31] showed that minimal use of injections reduces the risk of infection through parenteral route and also reduces the cost of treatment. The percentage of drugs prescribed from the hospital's essential drug list was 85.6%. This is comparable to the overall essential medicine list prescribing adherence of 88.0% found in a systematic analysis of prescribing indicators at primary health care centers within the WHO African region [22]. Non-optimal use of Essential Medicines List (EML) has been attributed to a number of reasons. These include lack of effective distribution of EML among prescribers, inadequate sensitization of prescribers and inadequate enforcement mechanisms [22]. Availability of essential drugs has been shown to be significantly associated with patients' utilization of health services and their satisfaction [21]. In this study, it was observed that 69.5% of all drugs prescribed were actually dispensed at the pharmacy. This finding is comparable to the findings in an earlier Ghana study [21], but lower than the 73.4% rate obtained in a survey of the pharmaceutical sector of Ghana [32]. The percentage of drugs actually dispensed is an important indicator of availability of medicines and thus can be used to measure the efficiency of any health facility's supply chain of drugs. The findings in this study, suggests that, there might be potential challenges with the hospital's drug supply chain. This could be due to the unavailability of drugs obtained from the National Health Insurance Scheme. Patients' knowledge on the correct dosages of their prescribed drugs was found to be 88.1%. This is high compared to the findings of some previous studies in Ghana that found 63.2% [21] and a range of 23-50.2% [32].

The study also found that patients' ability to recall prescribed doses correctly was significantly associated with the number of drugs per prescription. About 80% of all patients who received 6 or more drugs were unable to correctly repeat dosage schedules. This is consistent with the findings of a study in Nigeria [33], that found that as the number of drugs per prescription increased, the percentage of patients who could correctly recall their dosage regimens decreased significantly. Upper respiratory tract infections were the most common conditions for which antibiotics were prescribed in this study. In a study conducted in the United States, it was found that 1 in 3 antibiotic prescriptions were unnecessary and that in most out-patient facilities, the most inappropriate use of antibiotics occurred when they were applied in the management of common viral infections that do not need antibiotic treatment such as common cold, most coughs and most sore throats [34]. The Standard Treatment Guidelines (STG) are documented treatment strategies systematically developed to aid practitioners in choosing appropriate treatments for specific clinical conditions. The Ministry of Health, Ghana has been publishing a list of essential drugs with therapeutic guidelines since 1983 to aid the rational use of medicines in the country [12]. In this study, the assessment of adherence to the national STG among prescribers revealed that only 64.5% of all prescriptions were in conformity with the guidelines. The most probable reason for this low adherence is poor access and low use of the STG. Only (5.42%) of prescribers had either soft or hard copies of the guidelines. This finding is low compared to what was found in another primary health facility in Ghana [21], that found that about 77.8% of prescribers had access to the guidelines. The findings of this study could be a reflection of the general practice of prescribers in the country even though the guidelines are free and available both online and printed. The study was however associated with a few limitations. Biases due to inconsistencies in the drug supply chain, seasonality of diseases and the influence of the National Health Insurance Scheme were not assessed in this study. Also, there may be possible biases in prescriber behavior since they were aware their prescriptions were being studied. The study was conducted in a single facility and therefore findings cannot be generalized to all facilities in Ghana. Despite these limitations, this study has a number of strengths that make it an important contributor to the scientific literature on antibiotic prescriptions. The study is the first of its kind in the Central region of Ghana that uses the WHO rational drug use indicators to investigate antibiotic use in a primary health care facility. A larger sample size was used in this study compared to previous similar studies in Ghana so as to increase the reliability of estimates.

## Conclusion

The pattern of antibiotic prescriptions in this study was generally not satisfactory. In order to improve on both prescribing and patient care indicators, the hospital's Drug and Therapeutic Committee (DTC) should be strengthened to control antibiotic use. The hospital's DTC should regularly provide clinicians with up-to-date information regarding rational antibiotic use and the benefits both patients and the hospital will enjoy if evidence based medicine is practiced. Again, clinicians should be encouraged to obtain and use the STG. This study could provide baseline data for further studies that will investigate the reasons why there is polypharmacy and high antibiotic usage in this setting and why clinicians are reluctant to own and use the standard treatment guidelines.

### What is known about this topic

- High number of drugs prescribed per encounter;
- High antibiotic use by prescribers.

### What this study adds

- There is polypharmacy among prescribers;
- There is irrational use of antibiotics by prescribers;
- Patients' knowledge about their medications is significantly associated with the number of drugs per prescription, age and educational level.

## Competing interests

The authors declare no competing interests.
